# WNK1/HSN2 Mutation in Human Peripheral Neuropathy Deregulates *KCC2* Expression and Posterior Lateral Line Development in Zebrafish (*Danio rerio*)

**DOI:** 10.1371/journal.pgen.1003124

**Published:** 2013-01-03

**Authors:** Valérie Bercier, Edna Brustein, Meijiang Liao, Patrick A. Dion, Ronald G. Lafrenière, Guy A. Rouleau, Pierre Drapeau

**Affiliations:** 1 Department of Pathology and Cell Biology, Université de Montréal, Montréal, Québec, Canada; 2 Groupe de Recherche sur le Système Nerveux Central, Université de Montréal, Montréal, Québec, Canada; 3 Centre of Excellence in Neuroscience, Centre Hospitalier de l'Université de Montréal Research Center, Montréal, Québec, Canada; 4 Department of Medicine, Faculty of Medicine, Université de Montréal, Montréal, Québec, Canada; University of Minnesota, United States of America

## Abstract

Hereditary sensory and autonomic neuropathy type 2 (HSNAII) is a rare pathology characterized by an early onset of severe sensory loss (all modalities) in the distal limbs. It is due to autosomal recessive mutations confined to exon “HSN2” of the WNK1 (with-no-lysine protein kinase 1) serine-threonine kinase. While this kinase is well studied in the kidneys, little is known about its role in the nervous system. We hypothesized that the truncating mutations present in the neural-specific HSN2 exon lead to a loss-of-function of the WNK1 kinase, impairing development of the peripheral sensory system. To investigate the mechanisms by which the loss of WNK1/HSN2 isoform function causes HSANII, we used the embryonic zebrafish model and observed strong expression of WNK1/HSN2 in neuromasts of the peripheral lateral line (PLL) system by immunohistochemistry. Knocking down wnk1/hsn2 in embryos using antisense morpholino oligonucleotides led to improper PLL development. We then investigated the reported interaction between the WNK1 kinase and neuronal potassium chloride cotransporter KCC2, as this transporter is a target of WNK1 phosphorylation. *In situ* hybridization revealed *kcc2* expression in mature neuromasts of the PLL and semi-quantitative RT–PCR of wnk1/hsn2 knockdown embryos showed an increased expression of *kcc2* mRNA. Furthermore, overexpression of human KCC2 mRNA in embryos replicated the wnk1/hsn2 knockdown phenotype. We validated these results by obtaining double knockdown embryos, both for wnk1/hsn2 and kcc2, which alleviated the PLL defects. Interestingly, overexpression of inactive mutant KCC2-C568A, which does not extrude ions, allowed a phenocopy of the PLL defects. These results suggest a pathway in which WNK1/HSN2 interacts with KCC2, producing a novel regulation of its transcription independent of KCC2's activation, where a loss-of-function mutation in WNK1 induces an overexpression of KCC2 and hinders proper peripheral sensory nerve development, a hallmark of HSANII.

## Author Summary

Hereditary sensory and autonomic neuropathy type 2 (HSANII) is a rare human pathology characterized by the early loss of sensory perception. It arises from expression of autosomal recessive mutations confined to an alternatively spliced exon of the WNK1 (with-no-lysine protein kinase 1) serine-threonine kinase, which confers nervous system specificity. In zebrafish embryos, wnk1/hsn2 is expressed in the neuromasts of the posterior lateral line (PLL), a peripheral mechanosensory system of aquatic animals. Defects in the development of this system, both in the number of individual neuromasts and of the hair cells they possess, were observed upon knockdown of the wnk1/hsn2 isoform. We investigated interactions between the WNK1 kinase and the neuronal potassium chloride cotransporter 2 (KCC2) in the context of HSANII, as KCC2 has been implicated in regulating neurogenesis. WNK1 is known to phosphorylate KCC2, regulating its activity and possibly its expression levels. We found that kcc2 is expressed in mature neuromasts and observed an increased level of *kcc2* RNA in wnk1/hsn2 knockdown embryos. We suggest that the loss-of-function mutations in WNK1/HSN2 linked with HSANII lead to an imbalance in the levels of KCC2, deregulating its levels of transcription and hindering proper peripheral nervous system development.

## Introduction

Hereditary sensory and autonomic neuropathies (HSAN) are rare inherited neuropathies predominantly characterized by sensory dysfunction associated with variable degrees of autonomous and motor involvement. HSANs were first classified in five distinct types according to clinical presentation of symptoms as well as age of onset and mode of inheritance 1. These distinct categories were later confirmed by identification of causative mutations by genome linkage studies, revealing heterogeneity amongst HSAN types both clinically and genetically. HSAN type 2 (HSANII, OMIM#201300) is of autosomal recessive inheritance and is characterized by an early onset sensory neuropathy, causing patients to lack all sensory modalities in a strictly peripheral glove-and-stocking distribution leading to a diagnosis in the first two decades of life 2. Other characteristics include a loss of tendon reflex, skin ulceration, Charcot joint, and spontaneous amputations while excluding motor involvement 3,4,5. In addition, upon sural nerve biopsy in affected patients, a reduction in the number of myelinated fibers is observed as well as a slight decrease in the number of non-myelinated fibers 6,7. In the absence of evidence suggesting degenerative changes in the peripheral nerves, HSANII is believed to be non-progressive and has been argued as being due to improper development 5. Despite there being published cases of HSANII since the last century, the mechanism leading to this disorder is still not understood.

Mutations restricted to an intron within the *WNK1* (lysine deficient protein kinase 1) gene were found to be responsible for HSANII (location 12p13.33, gene/locus OMIM #605232). This sequence was at first attributed to a new gene-within-a-gene and named ‘HSN2’ for hereditary sensory neuropathy type 2 8 but it was later revealed to be an alternatively spliced exon of the serine/threonine kinase *WNK1*, nestled between exon 8 and 9 of the 28 exon gene 9. WNK1 (NCBI Gene ID: 65125; HGCN:14540) is one of four members of the with-no-lysine (K) kinases, characteristic among other serine/threonine kinases by a uniquely placed lysine involved in ATP binding. Each WNK member contains a well-conserved kinase domain and multiple coiled-coil domains as well as a host of proline-rich regions and potential SH3 domain binding-sites, pointing to an involvement in protein complex formation and modulation of signaling 10. A large section of the *WNK1* gene, including exon HSN2, has no reported motifs suggesting any particular function. However, the isoform including the HSN2 exon, termed WNK1/HSN2, has been found to be selectively expressed in the nervous system whereas other isoforms of the WNK1 kinase are quite ubiquitously expressed in the CNS and other tissues 9,10. Within the neuron, WNK1/HSN2 is also sublocalized differently being found in the axon and cell body while isoforms lacking the HSN2 exon are confined to the cell body 9.

All mutations in the HSN2 exon reported to date are loss-of-function mutations, producing an early terminated mRNA which leads to a truncated protein 7,11. While a WNK1 knockout has proved to be lethal in mouse embryos, suggesting an important role in early development, selective knockout of the HSN2 exon has not been attempted thus far 12. The functions of WNK1 in the nervous system are not well understood. This kinase has been reported to interact with KCC2, potassium-chloride cotransporter type 2 (*SLC12A5* gene for ‘solute carrier family 12, (potassium-chloride transporter) member 5’, NCBI Gene ID: 57468, HGCN:13818; 13, which is selectively expressed in neurons and participates in the regulation of the chloride gradient. WNK1 phosphorylation of KCC2 occurs in immature neurons but is absent in adult neurons 13,14, emphasizing a developmental role. Interestingly, KCC2 has been shown to regulate neurogenesis in the zebrafish (*Danio rerio*) spinal cord 15,16, which suggests it may also play a role in peripheral neurogenesis. We therefore used this simple model to investigate whether WNK1 is implicated, perhaps *via* KCC2, in the development of the peripheral nervous system of zebrafish.

## Results

### Wnk1 And Wnk1/hsn2 Are Expressed Throughout Embryonic Development

**Figure 1:**
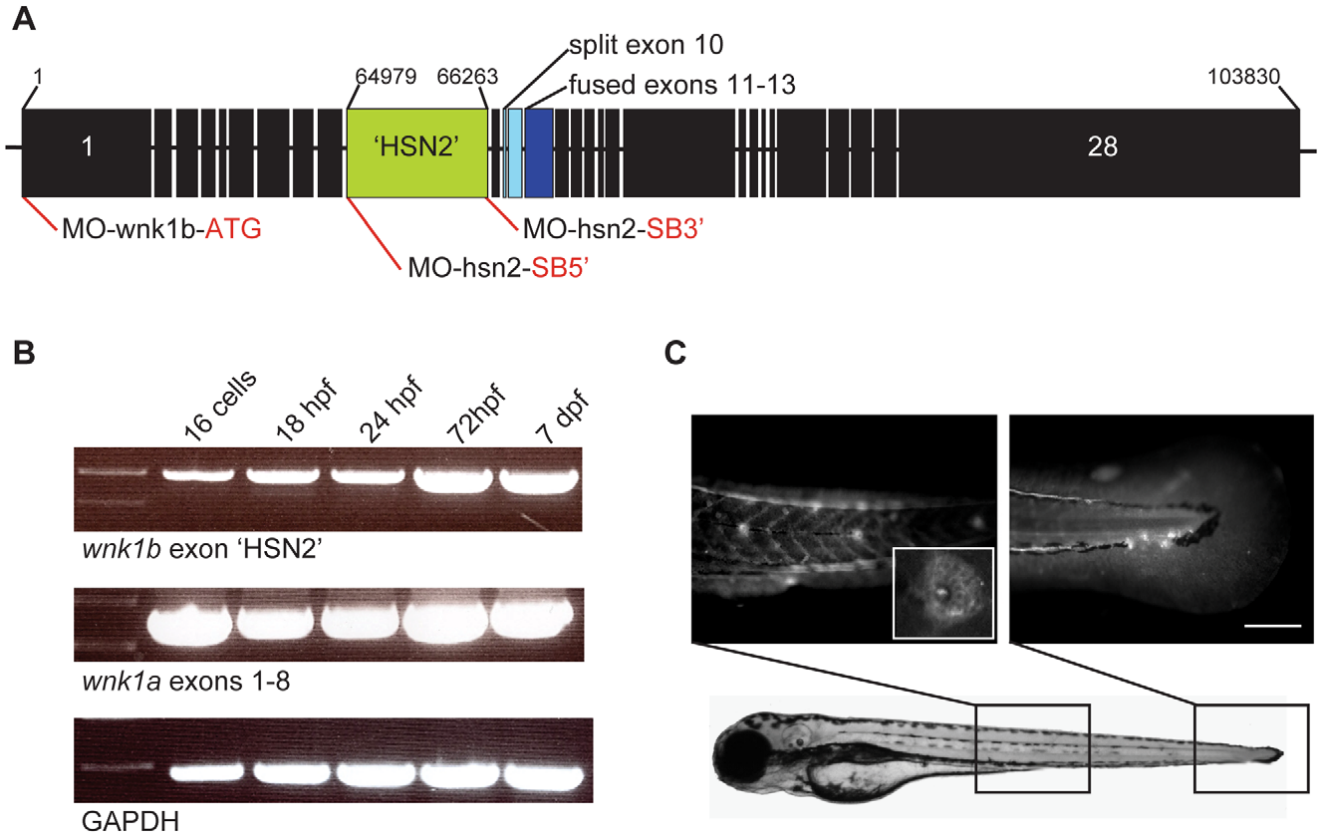
Expression of the WNK1 kinase in zebrafish embryos. A) Structure of the zebrafish WNK1 ortholog which conserved the HSN2 exon, *wnk1b*. The split exon 10 and fused exons 11–13 have been indicated on the sequence, respectively in pale blue and dark blue, as well as antisense morpholino oligonucleotide targets (red lines). B) Both copies of the zebrafish WNK1 ortholog, *wnk1a* and *wnk1b* are expressed early at the 16 cells stage and persist at 18, 24 and 72 hours post-fertilization (hpf) and until 7 days post-fertilization (dpf). The RT-PCR was done using primers set in the HSN2 exon for *wnk1b*, targeting a sequence spanning exons 1–8 for *wnk1a* and using as control the housekeeping gene *GAPDH*. C) Zebrafish WNK1/HSN2 from the *wnk1b* gene was detected in the neuromasts of the posterior lateral line by whole-mount immunohistochemistry using an anti-HSN2 antibody. The inset shows a closer view of a stained neuromast. Scale bar: 100 µm.

To investigate whether loss-of-function mutations in WNK1/HSN2 led to improper development of the peripheral nervous system, we used the zebrafish as it is a well-established model that is ideal for developmental biology since the first day post-fertilization roughly corresponds to the first trimester of mammalian development 17. It is also a model that has proven efficient in the study of functional genomics and pathogenesis of neurodegenerative disorders, with a relatively simple nervous system eliciting stereotyped responses 17,18,19,20. We first identified the zebrafish orthologs of the WNK1 kinase. Two separate loci were identified, named *wnk1a* (NCBI Gene ID: 100318736, ZFIN ID: ZDB-GENE-080917-49, chromosome 25) and *wnk1b* (NCBI Gene ID: 561159, ZFIN ID: ZDB-GENE-030131-2656, chromosome 4). These two genes were confirmed *via* Ensembl (Ensembl : ENSDARG00000078992). Only *wnk1b* conserves the HSN2 target exon (Figure 1A) and *wnk1a* appears to also be missing exons 11, 20, 21 and 22. Both copies have a split exon 10, and exons 11 to 13 of *wnk1b* are fused, which also appears in the *Xenopus laevis* ortholog sequence.

We next examined the developmental expression pattern of *wnk1b*. As the expression of the WNK1/HSN2 isoform had previously been assessed by Western blot in adult mouse tissue only 9, and there was no data available for its expression during embryogenesis. We first obtained an mRNA expression profile for both *wnk1a* and *wnk1b* by RT-PCR for zebrafish from the 16 cell stage to 7 days post-fertilization (dpf) (Figure 1B). Both orthologs were expressed early on with *wnk1b* expression increasing during the first few days whereas *wnk1a* expression was high from the start and maintained. The presence of the wnk1 and its wnk1/hsn2 isoform at the 16 cell stage (1.5 hpf) likely corresponds to a maternal transcript which leads early development prior to transcription of the zygotic genome at 3.5 hpf 21,22.

To localize the specific wnk1/hsn2 isoform within the nervous system, we performed whole-mount immunohistochemistry on 4 dpf zebrafish embryos using the previously described anti-HSN2 antibody 9. This revealed localization of the wnk1/hsn2 isoform (transcribed from the *wnk1b* gene) at the level of the posterior lateral line (PLL) neuromasts (Figure 1C) and not in the spinal cord. The wnk1/hsn2 protein was found within the two major neuromast cell types: hair cells and the support cells (inset, Figure 1C). This localization is consistent with HSANII to the extent that the neuropathy affects the peripheral sensory system and that the PLL is a peripheral mechanosensory system, albeit specific to aquatic animals.

### Knockdown Of Wnk1/hsn2 Perturbs Posterior Lateral Line Formation

**Figure 2:**
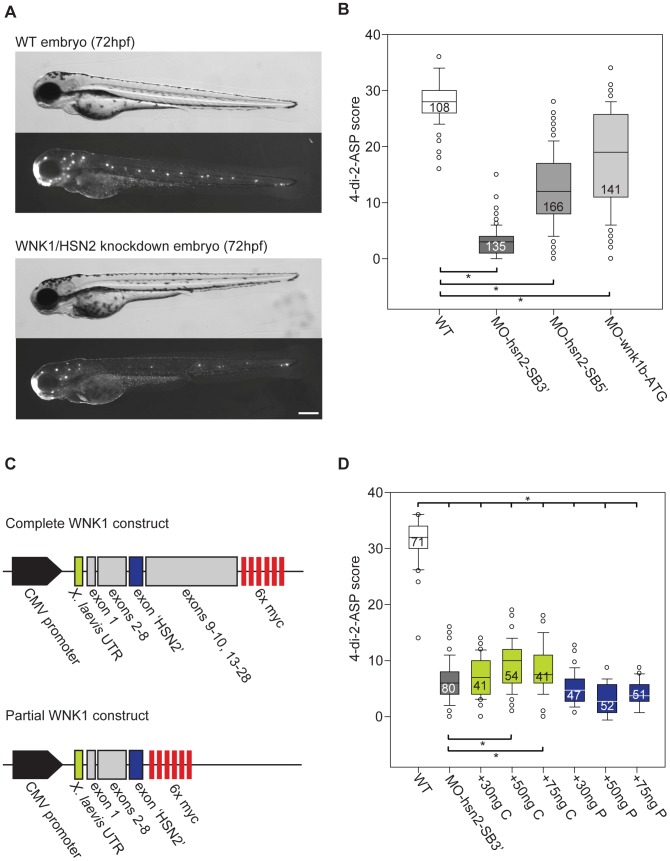
WNK1/HSN2 knockdown in zebrafish using antisense morpholino oligonucleotides (AMO). A) Knockdown embryos show no morphological phenotype, but reveal posterior lateral line defects (PLL) as observed under fluorescence with the 4-di-2-ASP vital dye when compared with non-injected WT embryos at 72 hours post-fertilization. The knockdown embryo presented is a representative result obtained from MO-hsn2-SB5′ injection. B) Each neuromast of the PLL observed with 4-di-2-ASP is assigned a score and totals for each fish is tabulated by condition and presented as a box plot, showing significantly lower scores for knockdown embryos when compared with WT. C) Two human sequence constructs of WNK1/HSN2 were designed for the rescue of the knockdown phenotype: a partial sequence containing exons 1-HSN2 and a complete construct containing exons 1–28 (but missing exons 11 and 12). D) 4-di-2-ASP score was assessed for embryos injected with MO-hsn2-SB3′ and concentrations of 30, 50 or 75 ng/µl of either partial or complete human WNK1/HSN2 constructs, revealing a partial rescue of the knockdown phenotype for embryos injected with 50 and 75 ng/µl of the complete construct. Scale bar: 100 µm.

In order to replicate the pathogenic loss-of-function of the WNK1/HSN2 isoform linked with HSANII causative mutations, we designed antisense morpholino oligonucleotides (AMO) targeting the start codon of *wnk1b* (AMO targets, Figure 1A). We also designed AMOs targeting the splice junction sites of exon hsn2 of the *wnk1b* gene, MO-hsn2-SB5′ and MO-hsn2-SB3′, respectively targeting the splice donor and splice acceptor sites. As the wnk1/hsn2 protein was detected by immunohistochemistry at the level of the PLL, we started by observing this mechanosensory system upon knockdown. Knockdown embryos for all three conditions were morphologically indistinguishable from non-injected animals at 72 hpf but staining of the lateral line with the fluorescent vital dye 4-di-2-ASP revealed defects in the formation of the PLL (Figure 2A). In order to quantify this phenotype, we attributed a score to each PLL neuromast depending on fluorescence to account for both their presence and composition, as was done previously 23. The scores were attributed accordingly: Full, fluorescent neuromast = 2 points; smaller or dim neuromast = 1 point; absent neuromast = 0 point. As the data was non-parametric, medians values were used to compare groups. In the wild-type non-injected fish (PLL neuromasts mid-body to tail, n = 108 embryos) we obtained a median value of 28.0 for the 4-di-2-ASP score. All three knockdowns, although with varying efficiency, revealed a significantly lower score, with median values of 3.0, 12.0 and 19.0 for MO-hsn2-SB3′ (n = 135 embryos), MO-hsn2-SB5′ (n = 166 embryos) and MO-wnk1b-ATG (n = 141 embryos) respectively, when compared with wild-type embryos (one-way ANOVA with Dunn's multiple comparison, Figure 2B). We further confirmed the specificity of the knockdown phenotype by rescuing it with wild-type human WNK1 mRNA. Two constructs were assembled for the human sequence: a complete construct spanning exons 1 to 28 (but skipping small exons 11 and 12 which were unavailable) and a partial construct composed only of exons 1 to HSN2 (*i.e.* lacking exons 9 to 28, Figure 2C) tested over a range of concentrations (Figure S1). The complete construct was co-injected with the most efficient AMO, namely MO-hsn2-SB3′ and significantly alleviated the PLL defect phenotype, without however bringing it back to wild-type level, at concentrations of 50 and 75 ng/µl (Figure 2D green boxes). The partial construct proved unable to rescue the knockdown phenotype when co-injected at similar concentrations with the AMO and thus confirmed the predicted loss-of-function of WNK1 following HSANII truncating mutations in the HSN2 exon 7,11 (Figure 2D blue boxes).

**Figure 3:**
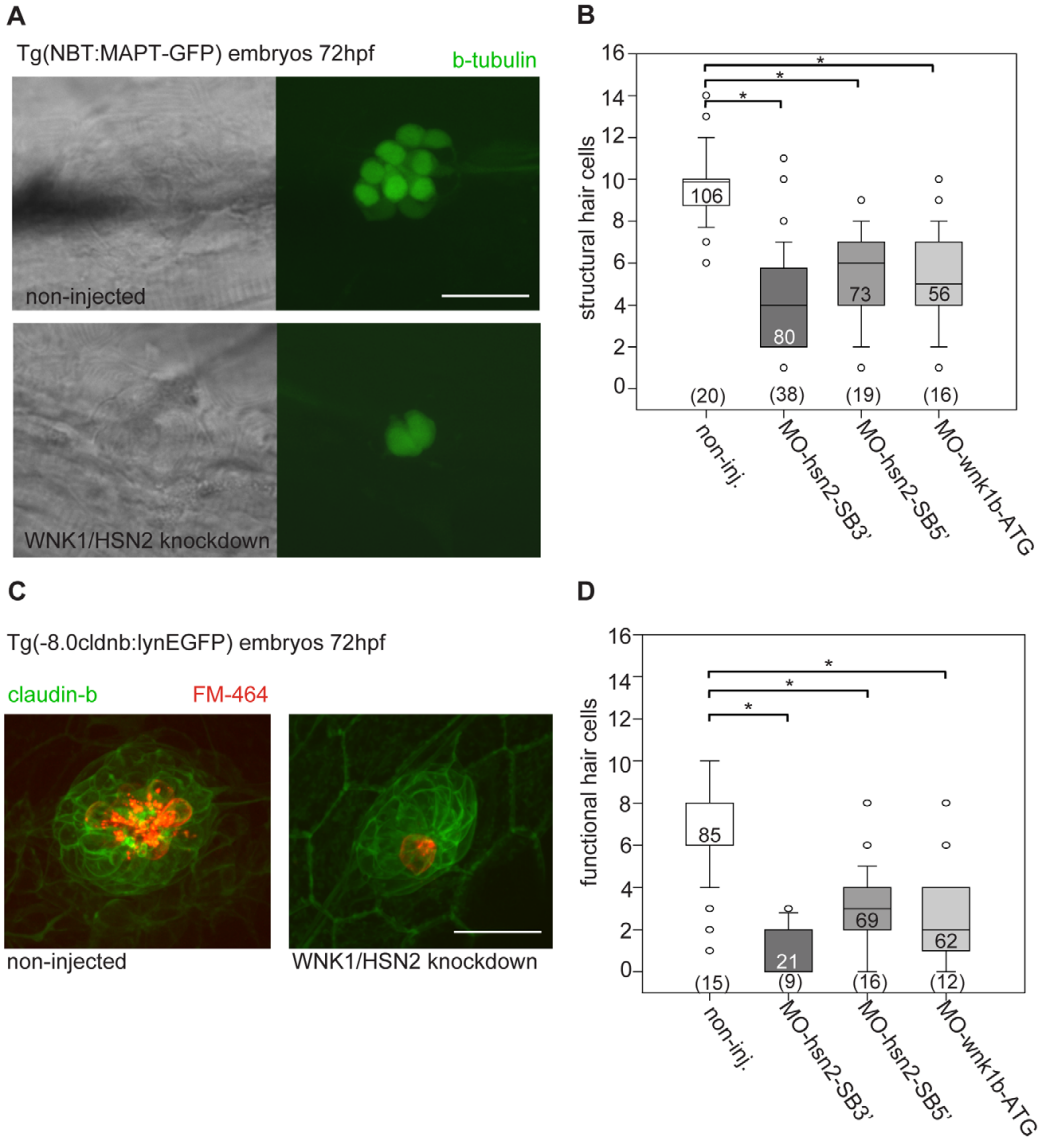
WNK1/HSN2 knockdown leads to abnormal neuromast development. A) The number of structural hair cells was assessed using transgenic embryos expressing GFP under the beta-tubulin promoter, revealing only the neuronal hair cells within PLL neuromasts. C) The number of functional hair cells was assessed by using transgenic embryos expressing GFP under the claudin-b promoter, rendering the neuromast fluorescent. The functional hair cells were revealed by incubation in the styryl dye FM-464FX, shown in red. B, D) Hair cells were counted for each PLL neuromast and totals were tabulated in box plots showing that WNK1/HSN2 knockdown embryos have a significantly lower number of structural and functional hair cells within their neuromasts when compared with non-injected embryos. The knockdown embryos presented in (A) and (C) are representative results at 72 hpf obtained from MO-hsn2-SB3′ injection. The number of neuromasts counted per condition is indicated in the boxes and the total number of embryos obtained per condition is indicated in parenthesis at the bottom of the box plots. Scale bar: 20 µm.

To further characterize the defects in PLL formation, we examined the structure of individual neuromasts by knocking down wnk1/hsn2 in transgenic embryos expressing GFP under the *Xenopus laevis* neuron-specific beta-tubulin promoter Tg(*NBT:MAPT-GFP*) 24 which allowed us to visualize structural hair cells based on the presence of beta-tubulin as revealed by expression of GFP (Figure 3A). An effort was made to count all PLL neuromasts of observed embryos, disregarding terminal neuromasts found at the tip of the tail because they arise from fragmentation of the primordium at the end of migration, and not by deposition of pro-neuromasts along the dorsal midline 25. The number of structural hair cells per PLL neuromast for all three types of wnk1/hsn2 knockdown embryos was significantly lower than for non-injected transgenic embryos, with median values of 4.0, 6.0 and 5.0 hair cells per neuromast for MO-hsn2-SB3′ (n = 80 neuromasts, 38 embryos), MO-hsn2-SB5′ (n = 73 neuromasts, 19 embryos) and MO-wnk1b-ATG (n = 56 neuromasts, 16 embryos) respectively, when compared with wild-type embryos which had a median value of 10.0 hair cells per neuromast (n = 106 neuromasts, 20 embryos; non-parametric distributions, one-way ANOVA with Dunn's multiple comparison; Figure 3B). We then confirmed this decrease by looking at the number of functional hair cells revealed by the vital styryl dye FM-464FX 26. Knockdown for wnk1/hsn2 was obtained in transgenic embryos expressing GFP under the claudin-b promoter Tg(*-8.0cldnb:lynEGFP*), which allows membrane labeling of primordium cells as well as neuromast hair cells and support cells 27. The knockdown and non-injected transgenic embryos were then incubated in FM-464FX and observed under fluorescence, where whole neuromasts would be seen in green (GFP) and hair cells, in red fluorescence (FM-464FX) (Figure 3C). While the number of support cells (labeled only in green) did not seem to decrease, the number of functional hair cells per neuromast decreased in a similar fashion to what had been observed for structural hair cells, where knockdown embryos had median values of 0.0, 3.0 and 2.0 hair cells per neuromast for MO-hsn2-SB3′ (n = 21 neuromasts, 9 embryos), MO-hsn2-SB5′ (n = 69 neuromasts, 16 embryos) and MO-wnk1b-ATG (n = 62 neuromasts, 12 embryos) respectively, when compared with non-injected embryos which had an median value of 8.0 hair cells per neuromast (n = 85 neuromasts, 15 embryos; non-parametric distributions, one-way ANOVA with Dunn's multiple comparison; (Figure 3D). The PLL defect phenotype thus seemed to leave support cells unaffected, suggesting a problem in neural maturation with only the hair-cell-fated neuromast progenitors failing to become functional.

### Wnk1/hsn2 Knockdown Embryos Overexpress Kcc2

**Figure 4:**
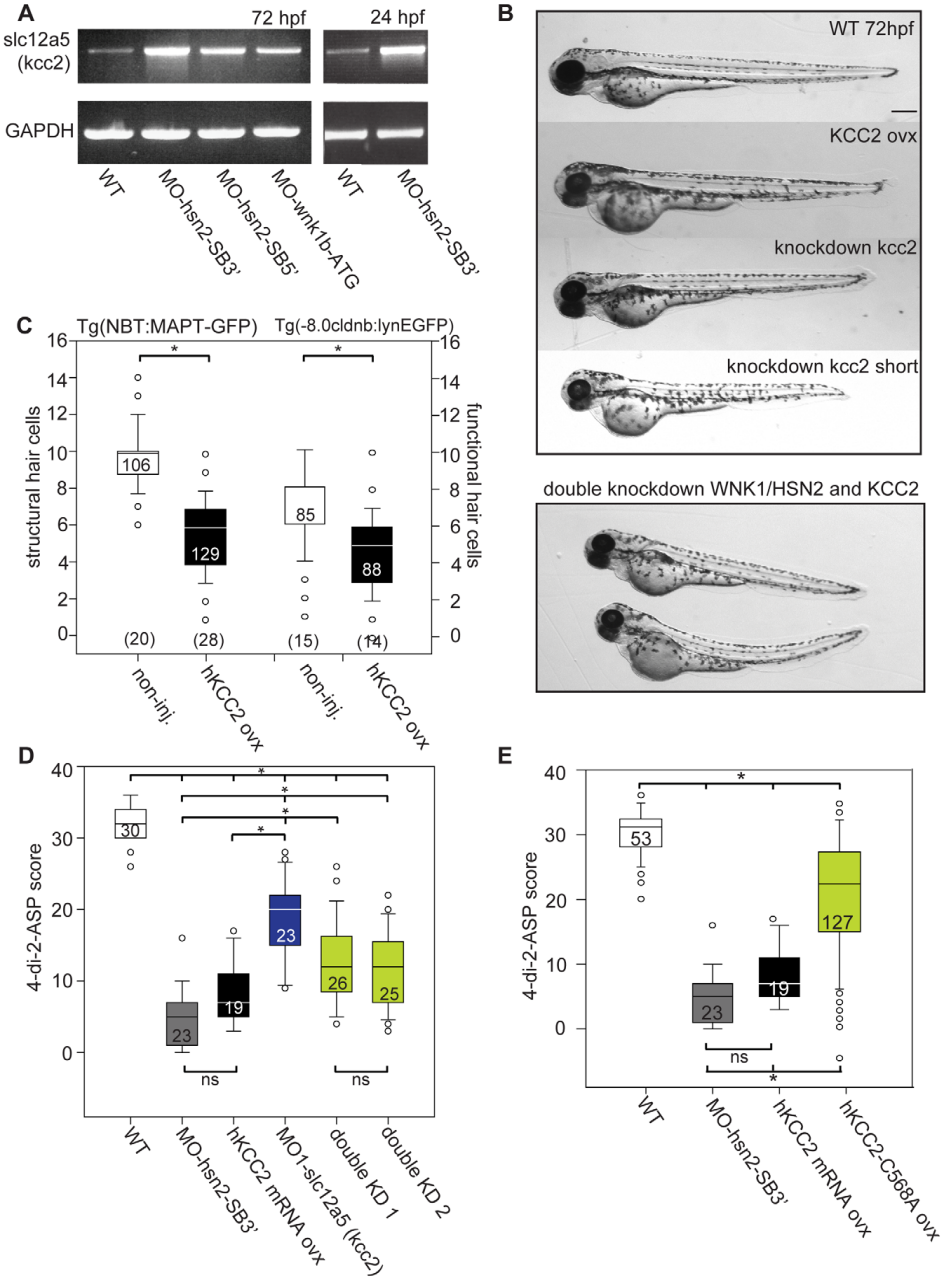
KCC2 is overexpressed in WNK1/HSN2 knockdown embryos. A) RT-PCR against *slc12a5* (coding for kcc2) shows higher levels of RNA in WNK1/HSN2 knockdown embryos when compared with WT at 72 hpf as well as at 24 hpf. We overexpressed human KCC2 mRNA in WT embryos (morphology presented in (B)) to validate this result and were able to replicate the WNK1/HSN2 knockdown phenotype as assayed by (C) the number of structural and functional hair cells in PLL neuromasts and by (D) the 4-di-2-ASP score. We also validated this result by obtaining a partial rescue of the WNK1/HSN2 knockdown phenotype by knocking down kcc2 using MO1-slc12a5 in WNK1/HSN2 embryos (double knockdown-KD experiments). The embryos lacking kcc2 have morphological defects and a lower 4-di-2-ASP score (D) due to their smaller length (B), but double knockdown embryos, which are also morphologically abnormal and smaller in size have a significantly higher 4-di-2-ASP score (D) indicative of a partial rescue. For (C) The number of neuromasts counted per condition is indicated in the boxes and the total number of embryos obtained per condition is indicated in parenthesis at the bottom of the box plots. For (D), the total number of embryos is indicated in the boxes. (E) Overexpression of inactive mutant KCC2-C568A mimics hKCC2 overexpression and WNK1/HSN2 knockdown phenotype by producing PLL defects as assayed by 4-di-2-ASP vital dye staining. This indicates that WNK1/HSN2 interacts with KCC2 and regulates its transcription independent of the cotransporter's activation. Neuromast scores were tabulated as previously done and presented as a box plot. The number of neuromasts counted per condition is indicated in the boxes. Scale bar: 100 µm.

The activity of the neuronal-specific KCC2 had recently been shown to be regulated by the WNK1 kinase, where phosphorylation decreased KCC2 activation 14. Because of this, we predicted the knockdown phenotype could increase the activity of the cotransporter, as it is usually downregulated by WNK1 kinases. In zebrafish, it has previously been shown that KCC2 (*slc12a5* gene, Ensembl: ENSDARG00000078187, ZFIN ID: ZDB-GENE-080707-1) becomes expressed in parallel with neuronal maturation. Its delayed expression allows a timely reversal of the chloride gradient and is essential for appropriate neuronal differentiation 15. In the absence of an antibody detecting kcc2 specifically in zebrafish, we examined mRNA levels by RT- PCR. At 72 hpf, when the PLL defect phenotype is visible in wnk1/hsn2 knockdown embryos, we indeed found a higher expression of *slc12a5* (Figure 4A). To determine if this overexpression was also a premature expression, which has been found to cause dendritic spine defects 28, we also looked at mRNA levels at 24 hpf and found early overexpression, shown for the most effective knockdown condition (Figure 4A).

### Human Kcc2 Overexpression Replicates The Pll Phenotype

To confirm that KCC2 is implicated in the wnk1/hsn2 PLL phenotype, we overexpressed human KCC2 mRNA in WT embryos as was previously described 15. At 72 hpf, embryos showed a normal morphology, though some animals had a shorter tail (Figure 4B). Upon labeling of the PLL with 4-di-2-ASP, we observed defects similar to the ones of wnk1/hsn2 knockdown embryos and confirmed a decrease in hair cell number, both structurally (Figure 4C) and functionally (Figure 4D, black boxes) upon overexpression of KCC2.

If the increase in *slc12a5* expression (coding for kcc2) following knockdown of *wnk1b* is indeed responsible for the loss of neuromasts, then we reasoned that knockdown of both *slc12a5* and *wnk1b* should rescue the phenotype. First we examined the effect of knockdown of *slc12a5* on its own using a previously described AMO (MO1-slc12a5, 29) for which knockdown is viable but leads to embryos with altered morphology, often exhibiting a shorter tail and curved spine (Figure 4B). Nonetheless, upon 4-di-2-ASP staining, these embryos had a structurally sound PLL, though with fewer neuromasts, which was probably due to their shorter length (score relative to WT, Figure 4D). Finally, we tested co-knockdown of *wnk1b* and *slc12a5* and observed a partial rescue of the PLL defect phenotype, as visualized with 4-di-2-ASP (Figure 4D, green boxes), confirming that the KCC2 cotransporter is implicated in the establishment of the wnk1/hsn2 knockdown PLL phenotype.

### Kcc2 In The Embryonic Posterior Lateral Line

**Figure 5:**
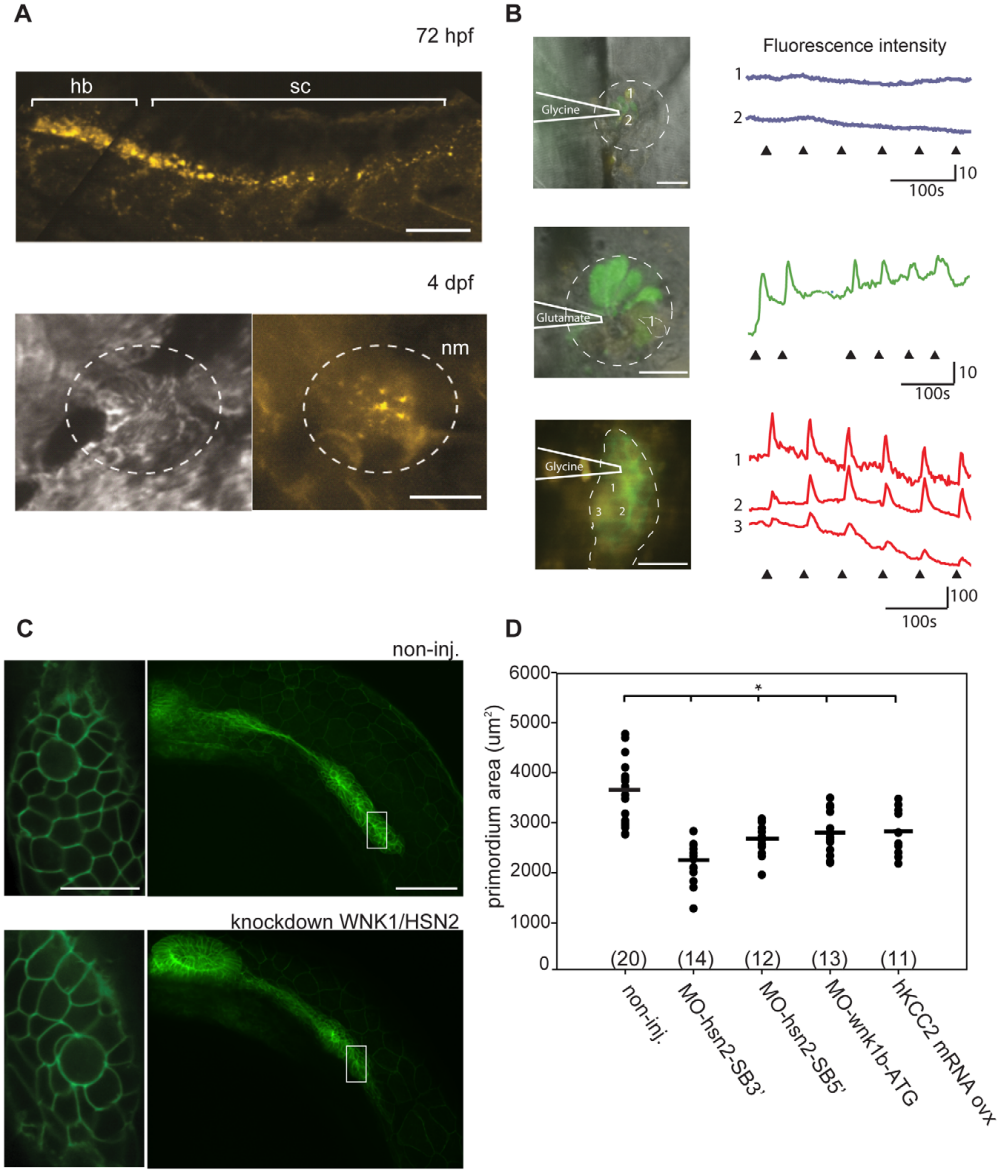
KCC2 is found in the embryonic zebrafish PLL. A) Presence of kcc2 in the zebrafish embryo was assessed by *in situ* hybridization against slc12a5 and reveals staining in the hindbrain (hb) and spinal cord (sc) at 72 hpf as well as staining in hair cells of a PLL neuromast (nm) at 4dpf. B) Neuromasts of 4 dpf transgenic Tg(*NBT:MAPT-GFP*) embryos expressing GFP (green) under neuron-specific beta-tubulin promoter (left upper and middle images) and primordium of a 2dpf transgenic Tg(*-8.0cldnb:lynEGFP*) embryos expressing GFP under the claudin-b promoter in the membranes of cells composing the primordium (left lower image) were labeled with the Ca^2+^ indicator Rhod 2-AM (deep red) to show that ionophoresis of glycine failed to evoke Ca^2+^ transients in 3–4 dpf neuromasts (right black upper traces) but did so in primordium cells (right red bottom traces). In contrast, neuromasts of embryos of equivalent stage respond to glutamate (right, green middle trace). The dashed line illustrates the primordium or neuromast region and the heavy lines illustrates the position of the pipet. C)Transgenic embryos expressing GFP under the claudin-b promoter, which labels the membranes of cells composing the primordium, were used to observe the size of this migrating group of PLL neuromast progenitors for both non-injected and WNK1/HSN2 knockdown embryos. Close-up image of the primordium cells shows no difference in organization between non-injected and WNK1/HSN2 knockdown embryos. D) The primordium area was measured on one side of the embryos and data was tabulated in a scatter plot which shows that WNK1/HSN2 embryos, as well as embryos overexpressing human KCC2, have a significantly smaller primordium area than non-injected embryos. The number of primordial measured is indicated in parenthesis at the bottom of the graph. The knockdown embryo presented in (C) is a representative results at 22 hpf obtained from MO-wnk1-ATG injection. Scale bars: (A) 100 µm and 20 µm for neuromast image, (B)20 µm, (C) 80 µm for full primordium and 20 µm for cell close-up.

As the presence of kcc2 has never been assessed in the zebrafish nervous system, we performed an *in situ* hybridization against *slc12a5*, revealing its expression in the hindbrain, in the rostral spinal cord and in neuromasts of 4dpf embryos (Figure 5A). Prior to kcc2 functional expression in the early zebrafish embryo, the chloride gradient is depolarizing due to the high chloride content 30. As a result, brief glycine application depolarizes the cells and evokes a Ca^2+^ transient 31. In contrast, we expected that if kcc2 is functionally expressed in neuromasts, the chloride content in its cells will be low and application of glycine will fail to evoke Ca^2+^ transients. We therefore loaded neuromasts with the Ca^2+^ indicator Rhod-2 AM and visualized their hair cells in 3–4 dpf transgenic Tg(*tub:MAPT-GFP*) embryos expressing GFP in axons. We observed that application of glycine onto these neuromasts (Figure 5C) failed to evoke Ca^2+^ transients (as shown in Figure 5B top image and traces; n = 4 neuromasts) whereas glutamate application did (Figure 5B middle image and traces), indicating that these neuromast cells were viable but unresponsive to glycine presumably due to the presence of kcc2 and a low intracellular chloride level. In contrast, application of glycine onto the primordium of 2dpf embryos visualized in Tg(*-8.0cldnb:lynEGFP*) 27 expressing GFP under the claudin-b promoter in cells composing the migrating primordium, progenitors of PLL neuromasts, and co-labeled with Rhod-2 AM evoked clear calcium transients (Figure 5B bottom image and traces; 6 cells in 2 primordia). This observation suggests a high chloride content in neuromast progenitor cells, the migrating primordium, much like the observations in spinal cord progenitors of equivalent stage zebrafish embryos 15,32. In summary, the lack of glycine evoked Ca^2+^ transients in neuromasts contrasted to their presence in the primordium and suggests that, as for spinal cord progenitors, kcc2 expression in neuromasts is functional and could be implicated in neural differentiation. This corroborates our previous results, showing an implication of KCC2 in WNK1/HSN2 knockdown phenotype, affecting the PLL.

### Wnk1/hsn2 Knockdown Affects Pll Progenitors

Previous experiments in zebrafish reported that overexpression of KCC2 leads to impaired neurogenesis by perturbing neuronal maturation 15,16. KCC2 has also been reported to be involved in mammalian neural development as a premature overexpression disrupts development of the neural tube by diminishing neuronal differentiation, leading to mouse embryos with a thinner neural tube and abnormal body curvature 33. To characterize the effect of KCC2 overexpression on PLL progenitors, we injected Tg(*-8.0cldnb:lynEGFP*) embryos and observed the primordium in live embryos in order to assess its size after departure from the cephalic placode but before deposition of the first pro-neuromast. It was not possible to count each individual cell as the primordium is a highly motile structure that is constantly reorganizing its cells. In an effort to quantify the observed effect, we embedded live animals in agarose while positioning them on their side, allowing us an optimal view of the primordium. We then captured images and measured the surface area of the primordium as revealed by GFP expression. WNK1/HSN2 knockdown embryos, as well as embryos overexpressing human KCC2, showed a significantly smaller primordium (Figure 5D). As the size of primordium cells and organization seemed conserved for all conditions (close-up, Figure 5C), we conclude that an overexpression of KCC2, whether it be by injection or induced by wnk1/hsn2 knockdown, resulted in a lower number of PLL progenitors. While the size of the primordium was reduced both in wnk1/hsn2 knockdown and in KCC2 overexpressing embryos, the organization and size of primordium cells seemed to be conserved (Figure 5C).

### Pll Defects Are Caused Independently Of Kcc2 Transporter Activity

KCC2 has been shown to have a role independent of its transporter activity, for example by influencing the development of dendritic spines through interaction with cytoskeleton protein 4.1 N 33,34, where loss 35 or premature expression 28 of KCC2 respectively induced abnormal morphology (lower number of functional spines) and an increase in dendritic spine density. Furthermore, phosphorylated KCC2 is found in neurons before the GABAergic response switch 36 and the implication of KCC2 in neuronal differentiation of the embryonic mouse neural tube was also shown to be independent of KCC2 activation and therefore independent of its chloride extruding function 33.

To determine whether the PLL defect phenotype resulting from KCC2 overexpression was due to its transporter function as a modulator of intracellular chloride concentration, we synthesized RNA for KCC2-C568A as this dominant-negative mutation has been shown to impair the chloride extruding function of the cotransporter and was used successfully as a control in the zebrafish spinal cord model 15. Here we show that overexpression of this inactive KCC2 mutant impairs proper PLL development in a similar manner to wild-type KCC2, as observed by 4-di-2-ASP staining (score, Figure 4E). We therefore suggest that loss of WNK1/HSN2 leads to an overexpression of KCC2 by a novel mechanism to regulate its transcription and that this overexpression impairs proper peripheral nervous system development in an activity-independent manner.

## Discussion

In this study, we show that WNK1/HSN2 truncating mutations associated with HSANII lead to a loss-of-function of this kinase isoform which causes developmental defects in a relevant structure in zebrafish, by impairing PLL formation. By immunohistochemistry, we showed localization of this isoform in the neuromasts composing the PLL and presented evidence of early mRNA expression for both zebrafish WNK1 orthologs, suggesting a role in early development. We showed that knockdown of *wnk1b* resulted in a defect of the peripheral nervous system manifested by fewer PLL neuromasts each containing fewer hair cells, measured both by their structural presence and by functional assessment, and that this specific phenotype could be partially rescued upon co-injection of wild-type human WNK1. The low efficiency of the rescue for the wnk1/hsn2 knockdown phenotype by human WNK1 RNA injection could be due to the fact that the human and zebrafish sequences are only 47% identical. Additionally, the constructs were made with no regard to endogenous patterns of alternative splicing as the HSN2 exon is poorly characterized and splicing data is sparse. It is possible that it is inadequately processed in zebrafish and can therefore only be of limited use in rescuing the knockdown phenotype. By injection of a partial construct mimicking truncating mutations in exon HSN2 we also confirmed the loss-of-function of this isoform in HSANII.

We found an overexpression of neuronal cotransporter kcc2 (*slc12a5* gene) in wnk1/hsn2 knockdown embryos, both at 72 hp and at 24 hpf. We replicated the PLL defect phenotype obtained in wnk1/hsn2 knockdown by overexpressing human KCC2 RNA in embryos, confirming a pathological link. To verify this link, we knocked down both wnk1/hsn2 and *slc12a5* by co-injecting AMOs, which partially rescued the PLL defect phenotype, thereby validating that this overexpression led to improper PLL development.

The *slc12a5* knockdown yielded morphologically abnormal embryos which still had a nicely developed PLL, though comprised of fewer neuromasts due to their shorter length. This observation is expected as KCC2 is known to be essential for proper neural development 15,36,37,38,39. Indeed, Kcc2 knockout in mice is embryonic lethal, causing defects in the development of the motor system leading to asphyxiation, as a reversal in GABAergic response necessary for proper neuronal maturation is never achieved 40. Knockdown of Kcc2 in neurons was also proven to compromise survival, by loss of its chloride extruding function 41. Both of these studies divulge a crucial for KCC2 in development and it is therefore not surprising to find abnormal morphology in embryos lacking a large proportion of their Kcc2. In contrast, overexpression of KCC2 leads to a neurogenic defect in the spinal cord of the early zebrafish embryo 15. Together, these observations support an important role of KCC2 regulation of the chloride gradient during development of the central nervous system.

We also demonstrated that kcc2 was localized to the peripheral nervous system (by *in situ* hybridization) at the level of the mature neuromast. Since KCC2 is known to be involved in neuronal maturation and proliferation, we assessed its effect on PLL progenitors. We found that embryos knocked down for wnk1/hsn2 (which overexpress kcc2) and embryos overexpressing human KCC2 had a smaller primordium, while maintaining normal cell size and organization, which led us to the conclusion that they contain fewer progenitor cells. However, the impact of this result on PLL formation is not clear. Indeed, the effect of primordium size on pro-neuromast deposition is a debated subject. It was previously reported 42 that ablation of up to two-thirds of the primordium leads to a defective PLL. This observed pattern is similar to the one observed in embryos lacking *lef1*, a major effector of Wnt signaling involved in the control of *cxcr4b* and *cxcr7b*, two chemokine receptors involved in PLL migration. The ablated and *lef1*-defficient primordium size is reduced after each deposition, and eventually disappears, having presumable run out of cells to deposit 42. Another study 43 has however discovered that a reduction of Notch activity gives rise to a smaller primordium, but the neuromasts deposited are of smaller size, rather than of regular size but fewer in number. This suggests a mechanism controlling the number of deposited pro-neuromast rather than one maintaining the size of deposits 43. The data was only acquired for the L1 pro-neuromast deposit and therefore it is possible that proliferative mechanisms taking place at the head of the migrating primordium 44, compensating for the deposits during migration, would be affected, leading to more severe defects further along in the PLL. While we did observe smaller primordia in wnk1/hsn2 knockdown and KCC2 overexpressing embryos, we can only suggest a possible role in progenitor proliferation, where as previously proposed, the deposition is triggered when the primordium reaches a threshold size 42. In this instance, progenitor proliferation would be affected at the placode and at the level of the mitotic head of the migrating primordium either by WNK1 as previously suggested (effect on proliferation, 45), or through interference of Notch signaling 43.

We therefore propose that loss of WNK1/HSN2 deregulates the levels of KCC2. Mechanisms controlling KCC2 expression are only beginning to be uncovered and due to a strikingly rapid turnover of the cotransporter at the cell membrane studies mostly looked at how activation influences transcription. For instance, it was previously shown that BDNF induces Egr4 expression, which rapidly activates the *KCC2b* promoter in immature neurons, increasing the expression of the KCC2 protein. In mature neurons, the BDNF/TrkB signaling pathway involving a downstream cascade implicating Shc/PRS-2 and PLC-gamma 46 was also found to reduce *KCC2* expression, in an activity-dependent manner 47. As for the downregulation of *KCC2*, it has been observed upon functional loss, where various stresses induced tyrosine dephosphorylation, resulting in decreased levels of KCC2 protein and mRNAs 48. These results also suggest KCC2 transcription could be controlled by its levels of activation, where rapid inactivation leads to a decreased production of mRNAs.

We were also able to mimic the PLL defect phenotype upon injection of an inactive KCC2 mutant (C568A) although it was achieved with statistical difference from both the wild-type embryos and the ones overexpressing hKCC2. These results suggest that a novel regulation of transcription, independent of KCC2 activation, may contribute to the phenotype. It will be therefore important to consider other roles of KCC2 with regards to its implication in neuronal development. For instance, this cotransporter has been reported to play a morphogenic role in dendritic spine formation 28 and is known to interact with cytoskeleton-associated protein 4.1 N 35). This interaction has also been shown to be diminished for the KCC2-C568A mutant where an overexpression could not replicate aberrant actin and 4.1 N patterns observed upon overexpression of WT KCC2 33. This could explain why we could not obtain a PLL phenotype as severe following injection of KCC2-C568A in zebrafish as what is observed when embryos overexpress KCC2 (Figure 4E). Since proteins like 4.1 N anchor the cytoskeleton to the plasma membrane 49, interaction with KCC2, possibly regulated by WNK1 phosphorylation, could prove crucial at this level. HSANII mutations found in the KIF1a kinesin 50 could also affect transport of cargo along the microtubules or unloading at axonal tips.

Previous studies localizing KCC2 mRNAs in rat have been unable to find staining in the primary sensory neurons of the dorsal root ganglia (DRG) and of the trigeminal nucleus presumably because these neurons have depolarizing responses to GABA, where the high intracellular chloride concentration is maintained by expression of NKCC1 38. However, another KCC family member KCC3 is also expressed in neurons, some interneurons, as well as in the spinal cord and in radial glia-like cells 51. This cotransporter has been studied in the context of hereditary motor and sensory neuropathy with agenesis of the corpus callosum (HMSN/ACC), where causative mutations have been identified in SC12A6 (coding for KCC3) 52. Truncating as well as loss-of-function mutations have been reported to cause mis-trafficking of proteins, decreasing their plasma membrane expression 53. This neuropathy is characterized by progressive sensory-motor deficits, where axonal swelling can be observed in patients. Since it is also found in radial glia-like cells, a role for KCC3 in migration and proliferation has been proposed 51. Additionally, KCC3 has homologous regulatory sites to the ones found on KCC2, phosphorylated by WNK1 (T991 and T1048 in KCC3) 13, but it has been reported to be unable to interact with cytoskeleton-associated protein 4.1 N 35. Both KCC2 and KCC3 deregulation could therefore be involved in improper development of the peripheral sensory nervous, with KCC2 leading to HSANII pathogenesis.

This article presents the first findings of the molecular basis for HSANII. The zebrafish model we have developed by use of AMO technology exhibited defects in a peripheral sensory system (the PLL) which were apparent during embryonic development, similar to the clinical description of HSANII. Motor defects were also absent upon observation of motor neurons in our wnk1/hsn2 knockdown embryos, which is also a characteristic of HSANII (Figure S2). Likewise, overexpression of KCC2 was shown previously to affect spinal cord interneuron populations but not motoneurons or intrinsic (Rohon-Beard) sensory neurons 15,16 and wnk1/hsn2 was not detected in the spinal cord, consistent with a selective role in sensory lateral line development. We hypothesized that the mutations identified in the HSN2 exon of HSANII patients, producing a truncated protein, would lead to a loss-of-function of this WNK1 and have validated this by using an AMO targeting the start codon of the wnk1 gene, blocking translation of all isoforms in the zebrafish embryos. However, by modifying the splicing patterns by use of the splice blocking AMOs, we confirmed that loss of the hsn2 exon was enough to induce the pathogenic phenotype. It is important to point out that this model is a transient one, due to the use of AMO technology, and that it does not provide full knockdown efficiency. It is therefore possible that the phenotype is not as severe as it would be in knockout animals and in future studies a zebrafish knockout could be obtained by genome editing. We have also identified a pathway involving the KCC2 cotransporter as a downstream target of the WNK1/HSN2 isoform. This cotransporter has been linked to neural differentiation and its regulation by WNK1 has previously been reported, but the interaction between them has not been investigated. Our results suggest the HSN2 exon is critical for normal development to take place and it would be very interesting to understand how its loss influences *KCC2* expression or affects WNK1 binding to the cotransporter.

Our results in the zebrafish indicate that KCC2 regulation by WNK1 is an important factor in promoting peripheral nerve development, which may be compromised in HSANII. Whether this is due to regulation of the chloride gradient and peripheral neurogenesis or in addition to a transport-independent KCC2 action in concert with related transporters remains to be determined.

## Materials And Methods

### Ethics Statement And Transgenic Animals

A colony of wild-type zebrafish (*Danio rerio*) was bred and maintained according to standard procedures 54. All experiments were performed in compliance with the guidelines of the Canadian Council for Animal Care and the *Comité de déontologie de l'expérimentation sur les animaux* (CDEA) of the University of Montreal. Embryos were anesthetized in 0.02% tricaïne (MS-222, Sigma) in Embryo medium prior to all experiments.

We used embryos from transgenic lines expressing green fluorescent protein (GFP) under various promoters as neuronal population markers.

Tg(*-8.0cldnb:lynEGFP*) : membrane-tethered EGFP(enhanced GFP) is under the claudinB promoter labeling the migrating lateral line primordial, the neuromast organs as well as the chain of interneuromast cells deposited during migration 27.

Tg(*NBT:MAPT-GFP*) : GFP is expressed under the *Xenopus laevis* neuron-specific beta-tubulin promoter 24.

### Genomic Structure Of Wnk1 Orthologs In Zebrafish

The sequence of human WNK1 was used to find homolog sequences in GenBank, leading to the identification of the Xenopus laevis ortholog of WNK1. We then used this ortholog sequence to search the zebrafish assembly using the BLAT genome browser from UCSC (http://genome.ucsc.edu/). The identified genomic sequence from zebrafish was then analyzed, and exons were identified through EST alignments or comparative genomic techniques. cDNA sequences were reconstituted based on the predicted exons and ORFs from the predicted cDNAs were used to derived the predicted peptide sequences. Exons were numbered from 1–28 and the HSN2 name was conserved for the target exon present only in the zebrafish *wnk1b* (chromosome 4). Orthologous protein sequences were aligned using CLUSTALW and amino acid identity/similarity was calculated using MatGAT program v2.01.

Exons 11 to 13 are fused, as is the case with Fugu and Tetradon, and exon 10 has been split in two smaller exons. The human WNK1 and zebrafish *wnk1b* orthologs are 47.4% identical and 56.8% similar along the full length of the proteins. We also compared the HSN2 exon sequence, but since the human putative exon 8b sequence 9 contains several frameshifting mutations, the chimp sequence was used instead for comparison purposes. Chimp and zebrafish *wnk1b* HSN2-like peptide sequences are well conserved, being 54.7% similar and 38.2% identical.

### Antisense Morpholino Oligonucleotides And Rna Injections

In order to obtain a knockdown of the wnk1/hsn2 isoform, we designed splice-block (SB) AMOs specific to the donor and acceptor splice sites of the HSN2 exon to interfere with pre-mRNA splicing (MO-hsn2-SB3′: 5′ - CGAGAACGAGTATTTCTAGGTACCA - 3′ and MO-hsn2-SB5′: 5′ - TGCAGTGACAATAACATACAGCATC - 3′). We also designed an AMO targeting the initiation codon of *wnk1b*, inhibiting protein translation from the only copy of the gene containing the HSN2 exon (MO-wnk1-ATG: 5′ - TTGGGATTTCCGATGACATCTTC - 3′) (Gene Tools, Philomath, OR).

To knockdown zebrafish kcc2 we used two AMOs targeting the initiation codon, the first of which had been previously used in another study (MO1-slc12a5 : 5′ - TGGATGTTGCATCTCCTGTGAACAT - 3′ from 29) and the second was designed according to the latest zebrafish genome assembly as a different target, confirming specificity of the resulting phenotype (MO2- slc12a5 : 5′ – CTCCTTTGATCTCCAGTGTCTGCAT- 3′).

Human KCC2 mRNAs (hKCC2) were transcribed from the Nhe1-linearized pGEMHE-KCC2 and pGEMHE-KCC2-C568A constructs using the T7 polymerase with the mMESSAGE Machine T7 Kit (Ambion, Austin, TX) as described previously 15. Both constructs were injected at the same concentration known to cause an overexpression phenotype 15,16.

Human WNK1 constructs were assembled in the pCS2 vector with a Cytomegalovirus promoter and a *Xenopus laevis* beta-glotine UTR region. A partial construct (containing exon 1 to HSN2) and a complete construct (containing exon 1 to 28, but missing exons 11 and 12) were both flanked with 6 myc tags. Exon 1 was amplified from human genomic, as well as exon HSN2, while sequences from exons 2–9 and 10–28 were obtained from clones (CF142377 and BC141881 respectively). mRNAs were transcribed from the KpnI-linearized plasmids using the mMESSAGE Machine SP6 Kit (Ambion, Austin, TX).

All AMOs and mRNAs were diluted in nuclease-free water (Ambion) with 0.2% FastGreen vital dye (Sigma) to judge of injection volume. Injections were performed in 1–4 cell stage zebrafish eggs using a Picospritzer III (Parker Hannifin, Cleveland, OH, USA) pressure ejector.

### Whole-Mount Immunohistochemistry

Immunohistochemistry was performed as previously described 15 against the HSN2 exon with the anti-HSN2 antibody previously used 9. The secondary antibody was a goat anti-rabbit Alexa Fluor 488 (Invitrogen). Imaging was performed using a compound fluorescence microscope (Nikon).

### Reverse Transcription–Pcr

All RT–PCR were performed using the Expand Long Template enzyme kit (Roche) against control housekeeping gene *GAPDH* performed with a 1∶2 cDNA dilution to avoid saturation. All samples were run on a 1% agarose gel containing ethidium bromide.

Total RNA from embryos of different developmental stages was extracted using the TRIzol reagent (Invitrogen, Carlsbad, CA) and cDNA was synthesized using the RevertAid H Minus First Strand cDNA Synthesis kit (Fermentas). Expression pattern of *wnk1a* and *wnk1b* was assessed using primer pairs amplifying the sequence between exon 1–8 and within exon HSN2 respectively.

wnk1a_exon1_f: CTACAAGGGACTGGATACGGAAACTAC

wnk1a_exon8_r: GAGCCTCGAGGATGGTCACTG

wnk1b_hsn2_f: GGGATGCCGGCTCAAAGATT

wnk1b_hsn2_r: TGATGGGACAAGGCAGGCTCGTG

In order to permit a comparison of levels of endogenous kcc2 in our various wnk1/hsn2 knockdown embryos, several precautions were taken in the RT-PCR protocol. Batches of injected embryos from each different group were obtained for the same clutch and staged. The same number of embryo was taken from each condition to perform total RNA extraction using the TRIzol reagent. RNA extraction as well as cDNA synthesis for each experiment was done in parallel, using master mixes whenever possible. Prior to this, we tested PCR parameters for *kcc2* primers using only wild-type cDNA in order to make sure the amplification would stay within the exponential amplification segment of the reaction in conditions where an overexpression occurs. The RT-PCR reaction was first tested using wild-type cDNA to establish the optimal condition for annealing temperature, elongation time as well as number of amplification cycles for comparison between conditions using the specific primer pair targeting endogenous kcc2 (*slc12a5*). Forward primer (slc12a5_f): TTTCACCGAGGGCCACATTGACG. Reverse primer (slc12a5_r): TCCACCTCCACGCACAAGAAGGAC. All samples from each experiment, as well as the GAPDH controls were run on the same 1% agarose gel.

### *In Situ* Hybridization

Hybridization was performed using sense and antisense probes designed against the zebrafish ortholog of KCC2 to view endogenous localization of *slc12a5* mRNA. Embryos of 4dpf and 7dpf were processed for *in situ* hybridization using fluorescent FastRed as previously described 55 with minor modifications, allowing for conservation of the superficial lateral line structure.

### Lateral Line Staining

The lateral line was labeled using the vital dye 4-(4-diethylaminostyryl)-N-methylpyridinium (4-di-2-ASP, Invitrogen) diluted to 0.5 mM in embryo medium. Embryos were dechorionated and staged at 72 hpf then incubated in the solution for 30 minutes at 28.5°C. They were then washed 3 times 10 minutes in fresh embryo medium and anesthetized before imaging on an epifluorescence dissection microscope (Olympus) equipped with a Flea2 CCD Camera (IEEE 1394, Point Grey Research Inc. Richmond, BC, Canada). This protocol, adapted from 56,57,58 allows for visualization of full neuromasts, as the dye gets incorporated into hair cells as well as support cells during a longer incubation period.

FM-464FX (Invitrogen), a styryl dye fixable analog, was also used as a vital dye for labeling functional hair cells. Embryos were dechorionated and staged at 72 hpf, then incubated for 1 minute in a 5 µM solution made from a diluted stock in DMSO. Embryos were then washed in embryo medium and anesthetized before being imaged by confocal microscopy.

### Calcium Imaging

Calcium imaging experiments were done using live transgenic embryos. To visualize the lateral line primordium and the neuromasts we used the Tg(-8.0cldnb:lynEGFP) embryos and to visualize the innervations of hair cells, we used the Tg(NBT:MAPT-GFP) expressing GFP under the neuron-specific promoter. 2–4 dpf embryos were anaesthetized in 0.02% tricaine (MS-222, Sigma) diluted in Evans solution (134 mM NaCl, 2.9 mM KCl, 2.1 mM CaCl2, 1.2 mM MgCl2, 10 mM HEPES, 10 mM glucose, pH 7.8, 290 mOsm). The embryos were then embedded in 2% low-melting-point agarose (Invitrogen) and placed on their sides in the recording chamber. Membrane-permeable Ca^2+^ indicator dye Rhod-2 AM (Invitrogen/Molecular Probes) was dissolved in DMSO with 20% Pluronic (Invitrogen/Molecular Probes) to yield a 10 mM stock solution and further diluted in Evans solution to a final concentration of 1 mM, as described previously 31. A small volume of the Ca^2+^ indicator was then pressure injected (Picospritzer III, General Valve Fairfield, N.J., USA) into the primordium or to the neuromast hair cell. Recordings were performed at room temperature in the presence of tricaine to block movement related Ca^2+^ transients 59 (Ashworth and Bolsover, 2002) and started recordings 60 min after the dye injection. Evoked Ca^2+^ transients were acquired for 20 minutes at 0.5 Hz (Volocity software, PerkinElmer) by confocal microscopy. To evoke calcium transients, glycine (1 M) or glutamate (100 mM, Na-glutamate, Sigma) were ionophoresed (MVCS-02, npi, Tamm, Germany) from a fine glass pipette (20–30 MΩ) 31. Background-corrected images were analyzed off-line with Volocity software and average changes in Ca^2+^ levels within regions of interest were calculated as ΔF/F, which is the ratio between the fluorescence change (ΔF) and the baseline fluorescence before stimulation (F). The images of the primordium presented in Figure 5 C were created by overlaying images taken from the green (GFP) and red (Rhod-2 AM) channels, while a bright field image of the neuromasts was added to better illustrate the location of the structure.

### Confocal Microscopy And Analysis

Embryos were anesthetized in 0.02% tricaïne (MS-222) in embryo medium and embedded in 1% low melting point agarose. Imaging was performed on a Quorum Technologies spinning-disk confocal microscope (Quorum WaveX Technology Inc Guelph, On, Canada) mounted on an upright Olympus BX61W1 fluorescence microscope with water-immersion lenses. The setup was fitted with a Hamamatsu ORCA-ER camera and image acquisition was done with the Volocity software (Perkin-Elmer) and analyzed with the ImageJ software (NIH). Stacks were acquired at 1 µm thickness and assembled in ImageJ before analysis. Merged images were obtained in Volocity and exported as TIFF files to be used in figures. Images were resized, cropped and brightness was adjusted using Photoshop CS2 (Adobe), the figures were assembled in Illustrator CS2 (Adobe).

### Statistical And Data Analysis

Data was plotted and analyzed using the Sigma Plot 11 software (Systat Software Inc., San Jose, CA, USA) and statistical significance was determined using one-way ANOVA combined with the Holm-Sidak method of comparison (normal distribution) or using Kruskall-Wallis one way ANOVA on ranks combined with Dunn's method of comparison (non-parametric distribution). Non-parametric data are presented using medians, data ranges in a box plot diagram. Each box features a central line representing the median value, where the box itself delineates 25–75% of the data range and error bars represent 10–90% of the data range. Outlying data points are represented as circles outside the box. Significance was established at p<0.05.

## Supporting Information

Figure S1 Rescue experiment of WNK1/HSN2 phenotype with a wider spectrum of human WNK1/HSN2 RNA concentrations. 4-di-2-ASP score was assessed for embryos injected with MO-hsn2-SB3′ and concentrations of either partial (30, 50, 75, 100 or 150 ng/µl) or complete (of 30, 50, 75, 100, 150 or 200 ng/µl) human WNK1/HSN2 constructs. Higher concentrations of RNA from these constructs did not reveal a more potent rescue of the phenotypical defects on the PLL. (TIF)

Figure S2 Motor neurons of wnk1/hsn2 knockdown embryos are not morphologically abnormal. Wnk1/hsn2 knockdown embryos expressing GFP under the HB9 promoter (Tg(mnx1:GFP) from Flanagan-Street *et al.*, 2005) were obtained for all three AMOs conditions (A-non injected embryo, B- MO-wnk1-ATG, C- MO-hsn2-SB5′, D- MO-hsn2-SB3′). The primary motor neurons did not show any defects at 48 hpf, which further parallels the HSANII pathology, where patients have no motor dysfunction. (TIF)
