# Supplementary figures and images for: WNK1/HSN2 Mutation in Human Peripheral Neuropathy Deregulates KCC2 Expression and Posterior Lateral Line Development in Zebrafish (Danio rerio)
Source: PLoS Genet. 2013 Jan 3;9(1):e1003124. doi: 10.1371/journal.pgen.1003124 (PMC3536653; doi:10.1371/journal.pgen.1003124)

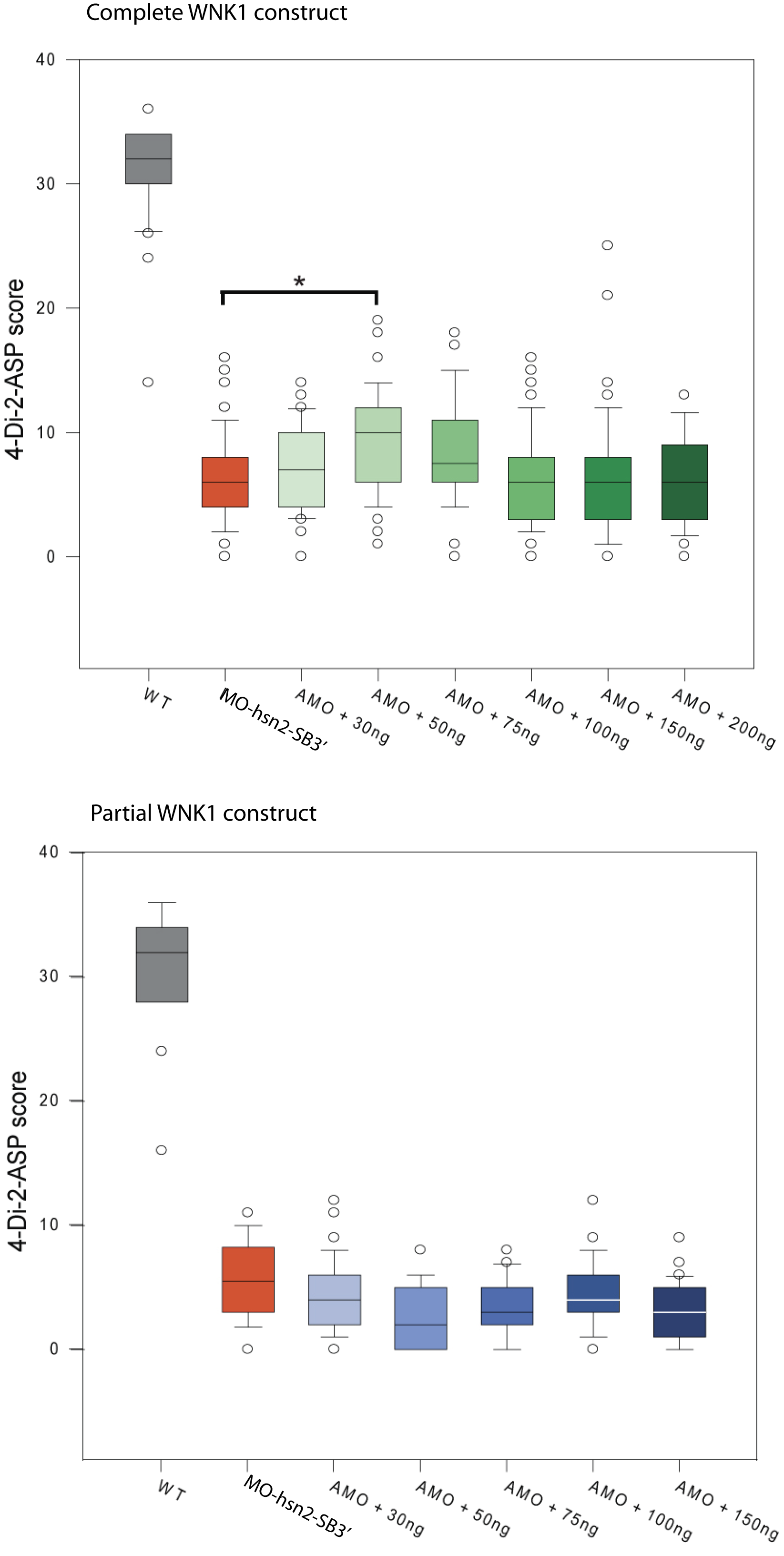

Supplement: Figure S1 — (TIF) [file pgen.1003124.s001.tif]

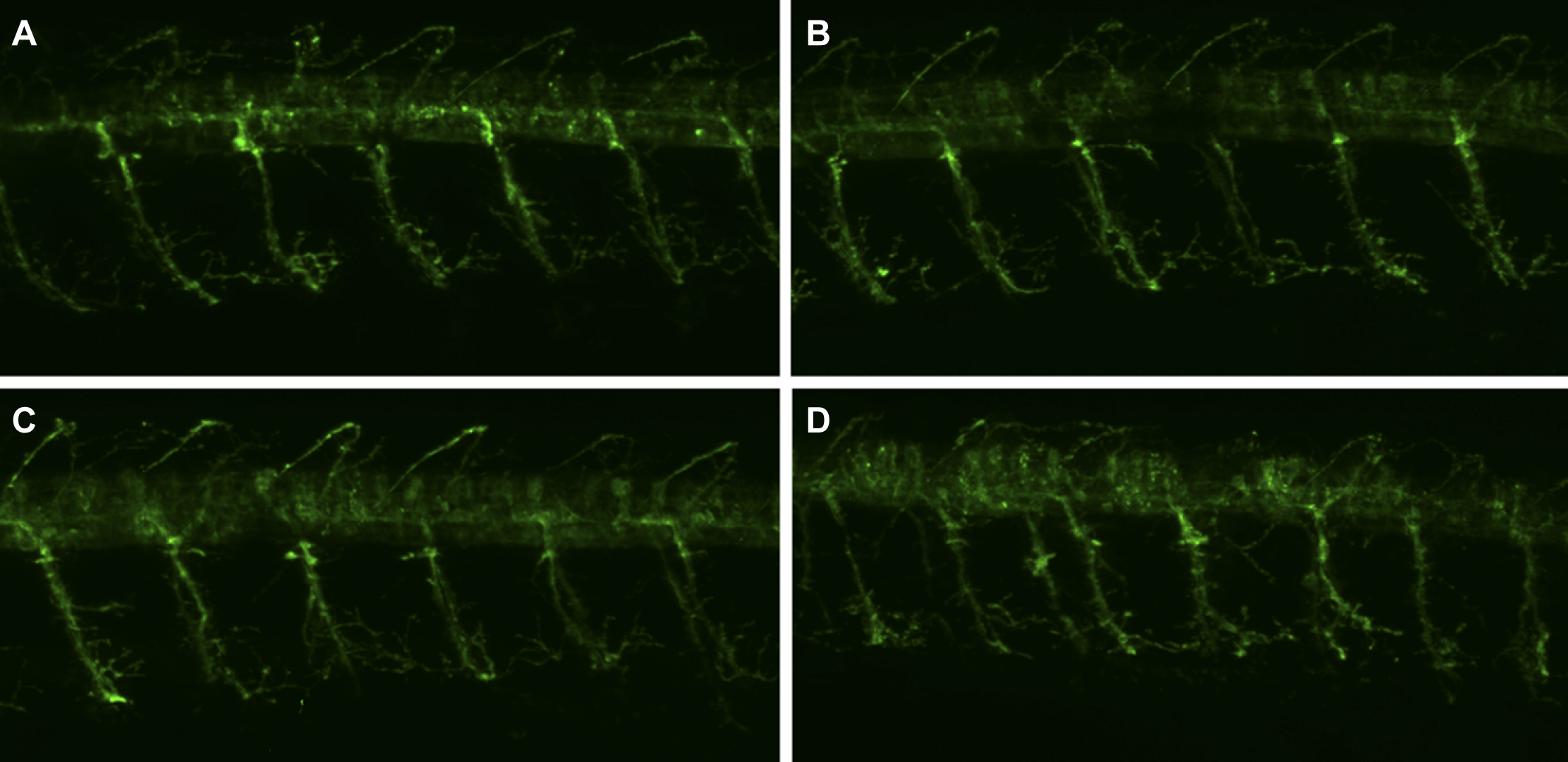

Supplement: Figure S2 — (TIF) [file pgen.1003124.s002.tif]
